# Indonesia: Epidemiological Profiles of Early Childhood Caries

**DOI:** 10.3389/fpubh.2019.00210

**Published:** 2019-08-06

**Authors:** Rosa Amalia, Fania Chairunisa, M. Fahmi Alfian, Al Supartinah

**Affiliations:** ^1^Preventive and Community Dentistry Department, Faculty of Dentistry, Universitas Gadjah Mada Yogyakarta, Yogyakarta, Indonesia; ^2^Pediatrics Dentistry Department, Faculty of Dentistry, Universitas Gadjah Mada Yogyakarta, Yogyakarta, Indonesia

**Keywords:** early childhood caries, Indonesia, caries, policy, children

## Abstract

The Indonesia government has succeeded in achieving national health development targets and has invested heavily in public health. Many positive results have been achieved, which indicate an increasing number of school-aged children free of caries and a decrease in caries experience scores. However, result of previous studies on early childhood caries (ECC) in pre-school children showed high prevalence and severity. Understanding the link between the epidemiology of the ECC and components of health development is critical for formulating appropriate actions. The purpose of this study is to provide a comprehensive review of the epidemiology of ECC in Indonesia based on the results of the national basic health surveys. The complementary data describes access, utilization and profile of oral health personnel in Indonesia.

## Introduction

Dental caries affects people of all ages and remains the major problem of dental health among children worldwide (1). For preschool children, early childhood caries (ECC) defined as the presence of one or more decayed (non-cavitated or cavitated lesions), missing (due to caries) or filled tooth surfaces in any primary tooth in children <71 months of age (2). ECC is highly prevalent (3), associated with multiple risk factor (4), and reduces the quality of life (5). If ECC treatment is delayed, the condition of the child worsens and becomes harder to treat, increasing the treatment cost.

ECC is also highly prevalence in preschool children living in developing countries like Indonesia (6). Every 5 years, the Indonesian government conducts basic health surveillance. There are surveillance reports on oral health and caries in children for the years 2007, 2013 and 2018 (7–9). Unfortunately, direct comparison of data is difficult because the reporting format is not in a same manner for each year. This study will therefore be limited to a comprehensive review of the available data on ECC accessible from the basic health surveillance reports, and data from the scientific publications on ECC in Indonesia. Situational analysis of oral health services in Indonesia will also be explored. This description is intended to be a highlight of the factors associated with ECC in Indonesia that may facilitate the review of a national oral health policy for Indonesia.

## Analysis On Policy Options and Implications

### Epidemiological Data Analysis on ECC in Indonesia

Table 1 presents an overview of the prevalence of ECC and possible risk factors in Indonesia extracted from the basic health surveys conducted in 2007, 2013, and 2018 (7–9). This table is quite revealing in several ways. It is apparent from the table that more than half of children aged 5 years were experiencing oral health problem. Unfortunately, those who got treatment were only 9.5%. The data also looks unsatisfactory since only 2% of the children received treatment of fillings or extraction. The most disappointing results to emerge from the national survey is that a high prevalence of ECC and poor behavior on brushing teeth properly.

**Table 1:** Oral health data pertaining to preschoolers from 2007 to 2018.

**Variables**	**2007**	**2013**	**2018**
**PERCENTAGE OF CHILDREN EXPERIENCING ORAL HEALTH PROBLEMS (PARENTS' PERCEPTION)**			
Age <1 years	1,1	1,1	NA
Age 1–4 years	6.9	10.4	NA
Age 3–4 years	NA	NA	41.1
Age 5	NA	NA	57.9
**PERCENTAGE OF CHILDREN WHO CLAIMED TO HAVE RECEIVED DENTAL CARE (PARENTS' PERCEPTION)**			
Age <1 years	28,1	36.9	NA
Age 1–4 years	27.4	25.8	NA
Age 3–4 years	NA	NA	4.3
Age 5	NA	NA	9.5
**PERCENTAGE OF CHILDREN WHO CLAIMED TO HAVE RECEIVED MEDICATION FOR DENTAL PROBLEMS (PARENTS' PERCEPTION)**			
Age <1 years	83,0	NA	NA
Age 1–4 years	93.0	NA	NA
Age 3–4 years	NA	NA	39.8
Age 5	NA	NA	48.9
**PERCENTAGE OF CHILDREN WHO TO HAVE RECEIVED FILLINGS OR AN EXTRACTION (PARENTS' PERCEPTION)**			
Age <1 years	10.9	NA	NA
Age 1–4 years	9.7	NA	NA
Age 3–4 years	NA	NA	0.8
Age 5	NA	NA	2.0
**PERCENTAGE OF RESIDENTS WHO CLAIMED TO HAVE BRUSHED THEIR TEETH PROPERLY (PARENTS' PERCEPTION)**			
Age 3–4 years	NA	NA	1.1
**AVERAGE SCORE OF DMFT**			
Age 3–4 years	NA	NA	6.2
Age 5 years	NA	NA	8.1
**PERCENTAGE OF CHILDREN WITH CARIES FREE**			
Age 3–4 years	NA	NA	19.0
Age 5 years	NA	NA	9,9

*NA, Not available*.

*Source: Ministry of Health Republic Indonesia (7–9)*.

However, a comparison of the data could not be conducted because of inconsistencies in the measures of the oral variables used for the surveillances. ECC severity using the dmft index, was only conducted in 2018. Also, the data on the dmft was not disaggregated. Data on the proportion of preschoolers who brush their teeth properly was only available in 2018. Despite this limitation, the table highlights a few important issues. First, it suggests that prevalence of ECC is increasing over the study period, and second, it suggests that children age 3–4 years contribute significantly to the high prevalence of ECC in the country. Caries severity also appears to increase by almost a third between the ages 3–4 and 5 years.

Publications on ECC in Indonesia has been limited in scope. Most of them were conducted in cities with few participants. In 1992, a study on preschool-aged children in the Jakarta area showed an ECC prevalence of 85.17% (10). Other research on ECC conducted in Jakarta in 2001 and 2008 reported prevalence of 81.2% (11) and 80.95% (12), respectively. The study in 200l was conducted in children 3–5 year old while that in 2008 was conducted in 3 year olds. The studies suggest that the prevalence of caries increased with age. Lower prevalence were reported in studies conducted in West and East Java; in West Java ECC prevalence was 70% (13) while it was 42.9% or children aged 3 years in East Java. The prevalence of severe ECC was 57.1% in East Java (14). Caries prevalence of 94.3% was reported in 4–6 year old children in Yogyakarta (15), and a prevalence of 100 and 88.5% reported in 5-year-olds resident in South Kalimantan and North Sulawesi, respectively (16, 17). Identified risk factors for ECC studies in Indonesia include low maternal education (12, 15), poor maternal knowledge of oral health (13, 16), and consumption of cariogenic foods, which had a significant relationship with the severity of ECC (12). Children who do not brush their teeth regularly from an early age also had high risk of ECC (15).

### Socio-Demographic Context

Indonesia is an archipelago state which is located between two continents (Asia and Australia) and two oceans (Indian and Pacific). The width of land area in Indonesia is around 1,916,862.2 km^2^, while the width of its sea is about 3,257,483 km^2^. Indonesia is a country with the fourth largest population worldwide (18). Indonesian people are comprised of many ethnics and cultures. There are more than 700 local languages and dialects in the daily life of its people (19).

It is evident that variations in caries prevalence among children are associated with multiple factors, including ethnicity and culture. Results of national basic health surveys in Indonesia also show that prevalence and severity of oral disease varies across ethnic groups. Yearly, the highest average caries index is found on the island of Borneo, followed by Java and Sulawesi. Factors like socioeconomic status, natural fluoride content in the water, and the culture of sweet food consumption have been suggested as causes of the high caries prevalence. unfortunately the data is presented in general for all ages, while specific data about ECC in children is not available. Interestingly, since the causes of caries are the same at all ages, it can be hypothesized that the variation in the prevalence of ECC across ethnic groups will not be much different.

### Health Policy Implications on ECC

Indonesia is classified as a country with lower-middle income population with increasing rate of national income per capita (20, 21). Despite this, 6.8% of Indonesians are living below the poverty line (22). To secure sufficient and sustainable health financing, The National Health Insurance (NHI) scheme was initiated to improve the access of all citizens to health. The scheme was started in January 2014 with the aim of covering about 250 million by 2019, thereby making it the world's largest social health insurance (23). In the Indonesia NHI scheme, oral health service is included as one of the benefit packages received by people. Benefit package for oral care in primary care including counseling, curative treatment (teeth restoration and minor surgery) and dental emergency care (24). This NHI package is valid for all ages, including children. The package also includes treatments for caries in children, including preventive restoration of fissure sealants. Unfortunately, to date, there is no detailed data available regarding utilization by groups of children under 5 years of age and what types of care are received by children related to ECC.

Recently, Ministry of Health developed the oral health provision grand design for 2015–2030 with the goal of having a healthy Indonesia free of caries by 2030. The Healthy Indonesia Free Caries campaign roadmap is revised every 5 years. The Road map started with the issuance of the National Act Plan of Oral Health Service which focused on strengthening policy, resources, and oral health service delivery. Targets were set for caries control in 12 years old children, oral health program at Community Health Centers (CHCs) were standardized, and the role of school-based dental programs and community-based health efforts were strengthened (25). The grand design of oral health provision accompanied the emergence of national policy on oral health laid out in the Ministry of Health Decree No. 89/2015 which identified oral health as an integral part of general health (26). Oral health services for infants focus on maintaining the health of the oral cavity before the teething stage, up to the age of 12 months. Information on oral health is provided to the parents and other family members by (a) counseling them about the growth phase of primary teeth, the conditions that accompany the process of teething and related abnormalities or diseases that often occur in infants as well as (b) educating them on how to maintain the health of their infant's oral cavity before teething until the incisors grow fully. It is stated that oral health services for children under the age of 5 years (between 12 and 72 months) is not very different from that for infants (26). The synergy of oral health policy for pre school and school children is expected to be a good foundation for reaching the target because, in the first 6 years, parents are expected to get good education about caries prevention for children, and the next 6 years of oral health education are applied directly to the children.

It is worthy to note that recently, the government of Indonesia has implemented the decentralization policy in the health sector, under Law No. 23/2014 on Local Government, stated that local government has a responsibility to focus on the delivery of defined essential services (which include health) while the central government has the responsibility to determine the expected standard for the provision of services. Decentralization plays a part, with each authority having an important role in setting policy in its region; every region has a unique approach depending on its individual needs. Unfortunately, reports about the success of ECC prevention program that take advantage of the opportunity for decentralization have not been published.

**Figure 1:**
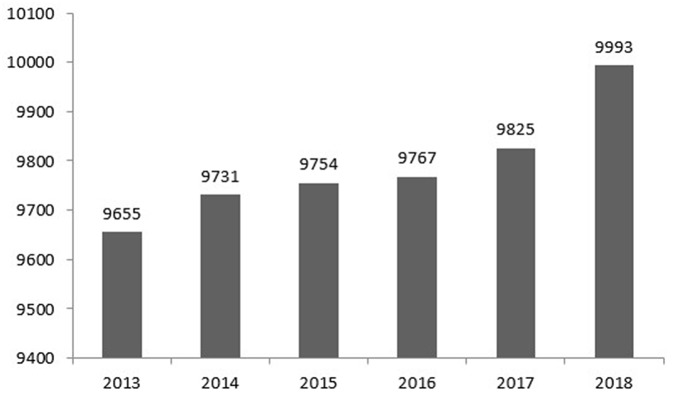
Number of community health centers in Indonesia (2013–2018). Source: Ministry of Health Republic Indonesia (18, 27–31).

**Figure 2:**
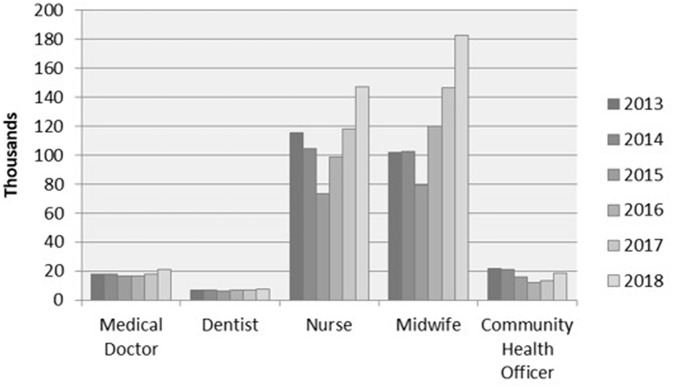
Number of health personnel in community health centers (2013–2018). Source: Ministry of Health Republic Indonesia (18, 27–31).

The number of community health centers and health workers in Indonesia has increased rapidly in the last few decades. Figure 1 shows the number of community health centers in Indonesia between 2013 and 2018. In 2013, this number stood at more than 9,600 units and then rose moderately to more than 9,700 units in 2014 and peaking at almost 10,000 units in 2018 (18, 25–31). Figure 2 compares the number of health workers working at CHCs between 2013 and 2018. Nurses and midwives account for the largest share of health workers, followed by medical doctors and community health officers (at ~20,000, respectively). Dentists accounted for the smallest share of health workers, with <10,000. Unfortunately, data on the national health profiles of dental therapists is only available for the years 2013 (10,150 persons) and 2014 (10,219 persons). The low number of oral health personnel and the unequal geographical distributions of existing oral health resources are constraints for the provision of oral healthcare. In 2018, 46.97% of CHCs did not have a dentist (18, 31).

Oral health personnel and CHCs have important role in the implementation of oral health policy regulations. Dental care has been offered to school children via the national school-based dental program (SBDP) for years. As part of the national school health strategy, SBDP are organized by community health centers located throughout Indonesia, and these aim to promote health by preventing oral disease and developing healthy behaviors. Community Health Centers also have responsibility to manage community-based dental programme where mothers and toddlers are the target of health programs including oral health. The issue of unsufficient number and unequal distribution of oral health personnel in Indonesia are quite worrying because the driving force for the dental public health program in CHC is expected to be the oral health personnel. It is evident that areas that have sufficient numbers of oral health personnel have been shown to have a school-based dental program that runs well and has a high rate of dental treatment to treat children's caries, and vice versa (32).

## Discussion

It is evident that the number of ECC studies carried out in Indonesia over the last 20 years is limited, yet in general the data generated consistently shows high prevalence. Methodological differences and confounding factors, especially socio-demographic influences, limit national comparisons of data on caries prevalence.

The increasing number of oral health personnel, along with the increase in the per-capita expenditure toward health services, does not seem to have had a significant impact. However, the decision maker (e.g., district health office) need to realize that the chosen strategic model to tackle ECC cannot be separated from the socio-economic context. For instance, since economic growth is a rapid process in Indonesia, consequently the consumption of sugar will be augmented. Therefore, dental caries in preschoolers can be expected to increase in coming years. In this case, successful cases of sugar restrictions policy in other countries could serve as examples of the importance of continuous oral health promotion (33).

Nevertheless, this approach is not without challenges. Sociodemographic variations can be a significant barrier, especially in low-income and low-education populations, and can have an impact on low awareness of the importance of oral health for children. This lack of awareness can be seen in the low rate of tooth brushing. This condition must be an alarm for policy makers because the current best practices for reducing the risk of ECC include twice-daily brushing with fluoridated tooth paste. Fortunately, due to policies in Indonesia, toothpaste is relatively affordable and must contain fluoride, so this should be an advantage in caries-prevention strategies. Following this, the role of parents is very important. This should be a concern because children's health development is very dependent on the health and well-being of their parents. The study concluded that some important health behaviors in parents, such as tooth brushing habits are important determinants of these behaviors in their young children. So promoting parent knowledge and attitude could affect their children oral health behavior and status (34).

Indonesia has had a series of policies regarding caries prevention strategies for children. The strength of this program is the direct involvement of parents for children under 6 years old, with parents at the center of counseling on how to maintain children's dental health. This program is community-based, with mothers as the targets. For children 6 years and older, the programs are directly applied and school-based. However, it is worth considering whether school-based dental programs that only focus on elementary education actually have a weakness, because children, at an early age (preschool, in kindergarten, or early childhood education), receive only limited information about good oral hygiene practices directly from oral health personnel. It is also important for oral health personnel to manage the ECC strategy for early childhood/kindergarten children conducted in schools, including conducting ECC risk assessments, teaching daily tooth brushing, and introducing the importance of regular dental visits.

Related to policy development at the regional level (decentralization), the role of the District Health Office is very important. Detailed directions or guidelines can be given to program implementers in the field regarding what should be conveyed to parents to prevent ECC, including the importance of avoiding sticky and sugary foods, not prolonging the use of bottles, and proper practice of oral hygiene. Decision-makers in each region will better understand the various approaches that can be attempted, depending on the specific conditions in each region. A policy of implementing a homogeneous strategy would not bring optimal results due to, for example, the presence of language and literacy barriers in remote and underdeveloped areas. Providing information using local languages or adapting modifiable procedures to cultures can help to overcome obstacles to understanding how to implement healthy behavior.

Despite limitations, there is a strength in the national policy implemented in Indonesia. NHI can be a guarantee of access to health services, reaching even families with low socio-economic status and children with special needs. It is important to note that dental health services at NHI also include preventive services, such as early detection, and preventive restoration, such as fissure sealants. However, parents often do not know that such services exist; they only know about services like restoration and tooth extraction (24). This understanding results in parents taking their children to the dentist only after the child has experienced pain (a toothache), which usually means the hole in the tooth is already deep.

It is important for oral health personnel to consistently and continuously conduct policy-maker advocacy regarding the consequences and preventive strategies for ECC. The national road map of the caries prevention strategy cannot only be targeted at school children, but must also be aimed at preschool children. The national target that considers only school children has even resulted in a lack of attention to ECC among oral health personnel themselves. This is such a concern because, nationally, there is no ECC program, and oral health personnel are not spread evenly. The high numbers of other health workers should be a resource that can be used to deal with dental diseases that occur in children.

## Actionable Recommendations

Taken together, the results of analysis on epidemiological data and oral health policy suggest following recommendations:

- National preventive program toward ECC should be prioritized including activity of prevention program in kindergarten or early childhood education. However, oral health programs for young children cannot rely solely on school-based and the decision maker must consider a reasonable range of alternatives to expand the provision of the program.
- Expanding the program via maternal care should be considered as a potential alternative. Integrating immunization programs with dental check-ups also could be another option, since this program is widely spread and accepted among citizen.
- Oral health training can be provided for other health workers, like midwives, to help them identify, and refer high-risk patients for subsequent care management in a dental clinic. Such programs should be actively delivered via early childhood education programs by involving teachers and health cadres via integrated healthcare posts.
- Under the NHI system, there is a potential scheme in implementing early screening for ECC for pre-schoolers. Children referred upon screening will be treated free of charge in CHCs.
- National data on caries should illustrate the prevalence for each of the provinces to indicate regional differences. If the decision maker seeks to develop an efficient and sustainable oral health system, the successes, and failures of the existing service model must be identified. The decision maker needs to realize that the chosen strategic model to tackle ECC cannot be separated from the socio-economic context.

## Conclusion

There is a necessity to review dental public health policies and develop effective strategies to prevent the epidemic of ECC in Indonesia. The key to success in the prevention and curative management of ECC lies not only in the availability of oral health personnel but also in a joint commitment with other parties. Further, advocacy to prevent healthcare problems among children is critical because this will have consequences for general health. In particular, policies that promote children's oral health as part of public health initiatives can help attract people's attention.

## Author Contributions

RA initiated the idea for the manuscript, wrote the initial framework, and edited the manuscript. FC, MA, and AS added materials according to their expertise. Each author critically reviewed the manuscript for its intellectual content.

### Conflict of Interest Statement

The authors declare that the research was conducted in the absence of any commercial or financial relationships that could be construed as a potential conflict of interest.
